# Disease in Bone and Its Detection by the X-Rays

**Published:** 1911-12

**Authors:** 


**Disease in Bone and its Detection by the X-rays.** By EDWARD H. SHENTON. Pp. xii, 72. London: Macmillan & Co. Limited. 1911.

It is not often that a book like this small monograph is met with, which is a plain, straightforward account of the author's own observations and the deductions that he draws therefrom. Consequently its short seventy odd pages, absolutely free from padding of all kinds, are of more value than many volumes of much greater size, and are full of useful suggestions to the radiographer and to the surgeon who relies on the use of the X-rays to help him towards a diagnosis.

The object which the author sets before him is " to record facts which radiographic experience makes him regard as fundamental in diagnosis," " facts which are not generally known, or surgeons would make more use of the X-rays, and not merely relegate them to the detection of coarse and obvious lesions."

The point on which the author lays the greatest stress is the difference in density in bone tissue in different diseases, and he lays down as a law that " generally speaking acute bone disease is made evident by increased transparency, and chronic disease by increase of opacity." This is well shown in radiograms of old cases of necrosis.

Again, if the outlines of a joint are blurred and indistinct in the radiogram, it is pathognomonic of tubercular disease, the pus and effused fluid in the joint in early cases, and the debris of bone tissue in addition in more advanced cases, effectually obscuring the outlines of the joint surfaces.

The consideration of syphilitic bone disease is purposely omitted, as the author's notes on the subject are not " ripe for publication." Further communications on the density of bone tissue in many chronic diseases, including alcoholism, are promised at a later date, which communications we shall look forward to reading with the greatest pleasure.

The book is very nicely got up, and the radiograms which illustrate the text are most excellently reproduced. This short work is to be cordially recommended to all radiographers and to others interested in the subject as one of the most valuable additions to X-ray literature which has appeared for a long time.

